# Supplementary material for: Seminoma and Embryonal Carcinoma Footprints Identified by Analysis of Integrated Genome-Wide Epigenetic and Expression Profiles of Germ Cell Cancer Cell Lines
Source: PLoS One. 2014 Jun 2;9(6):e98330. doi: 10.1371/journal.pone.0098330 (PMC4041891; doi:10.1371/journal.pone.0098330)
Supplement: File S1 — ZIP file containing DMRforPairs output for significant regions. Please start from the html files. (ZIP) [file pone.0098330.s008.zip › figures/27980.pdf]

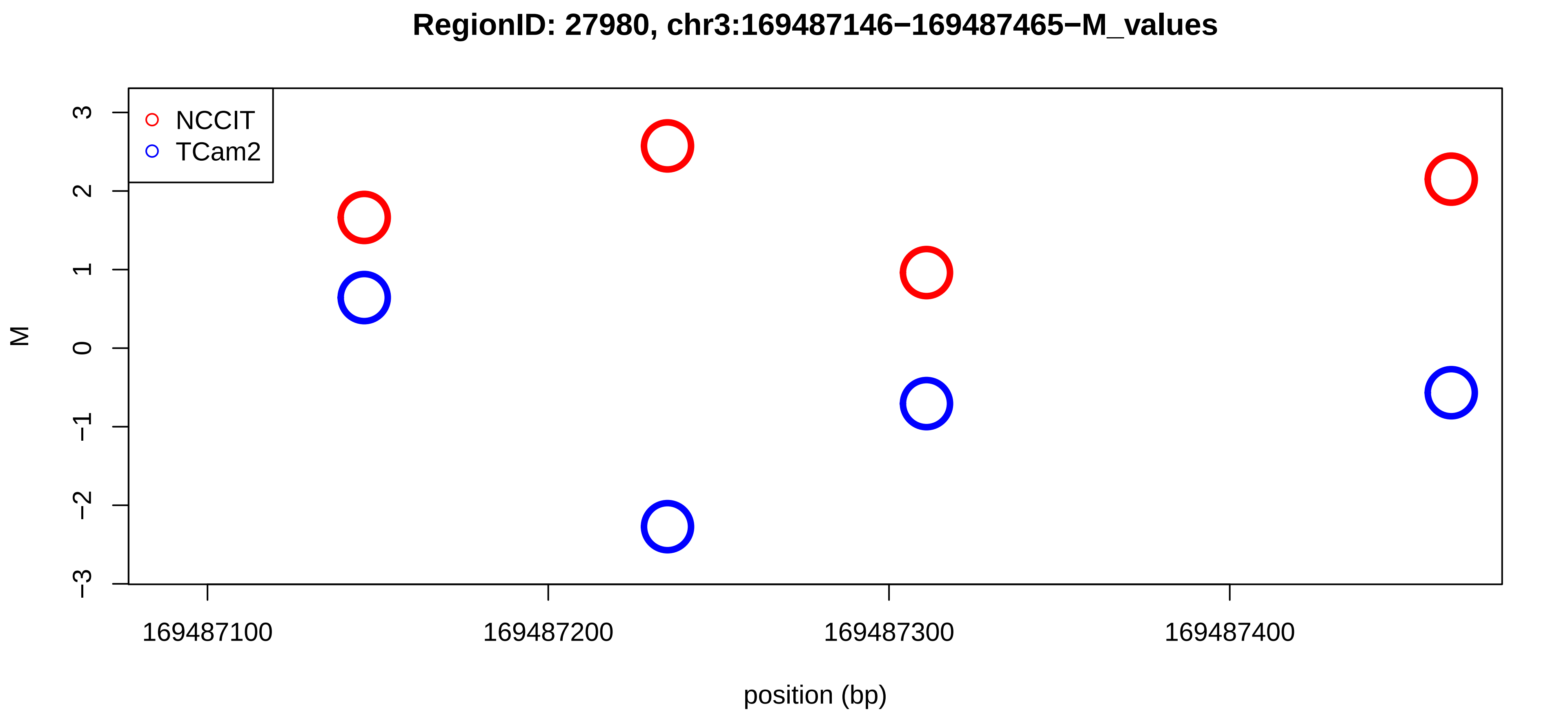
RegionID: 27980, chr3:169487146−169487465−M_values
NCCIT
TCam2
M
3
2
1
0
−1
−2
−3
169487100
169487200
169487300
169487400
position (bp)

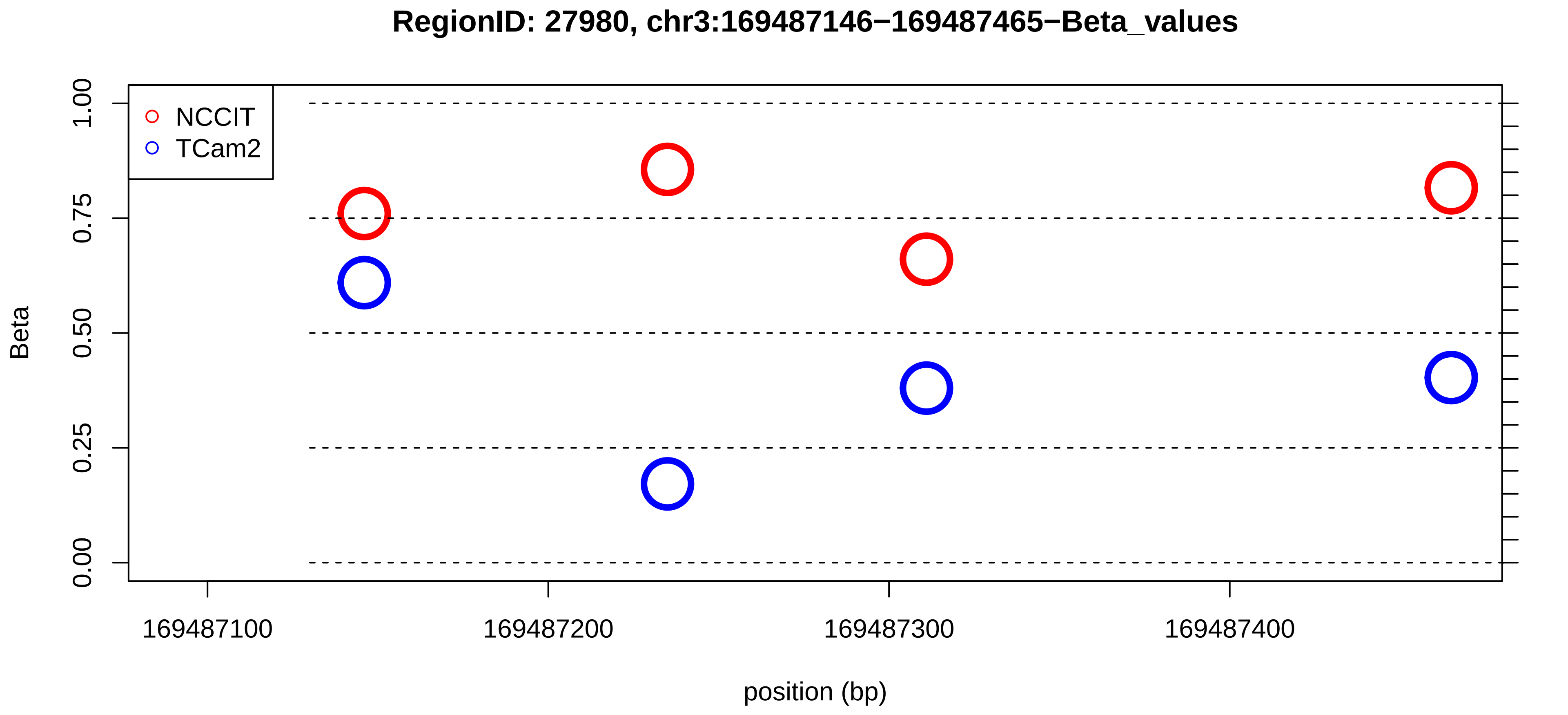
RegionID: 27980, chr3:169487146−169487465−Beta_values
NCCIT
TCam2
Beta
1.00
0.75
0.50
0.25
0.00
169487100
169487200
169487300
169487400
position (bp)
